# Medical treatment of heart failure with renin–angiotensin–aldosterone system inhibitors and beta-blockers in aortic stenosis: association with long-term outcome after aortic valve replacement

**DOI:** 10.1093/ehjopen/oeae039

**Published:** 2024-05-09

**Authors:** Johan Hopfgarten, Stefan James, Lars Lindhagen, Tomasz Baron, Elisabeth Ståhle, Christina Christersson

**Affiliations:** Department of Medical Sciences, Uppsala University, Akademiska sjukhuset, 751 85, Uppsala, Sweden; Department of Medical Sciences, Uppsala University, Akademiska sjukhuset, 751 85, Uppsala, Sweden; Uppsala Clinical Research Center, Uppsala University, Uppsala, Sweden; Uppsala Clinical Research Center, Uppsala University, Uppsala, Sweden; Department of Medical Sciences, Uppsala University, Akademiska sjukhuset, 751 85, Uppsala, Sweden; Uppsala Clinical Research Center, Uppsala University, Uppsala, Sweden; Department of Surgical Sciences, Uppsala University, Uppsala, Sweden; Department of Medical Sciences, Uppsala University, Akademiska sjukhuset, 751 85, Uppsala, Sweden

**Keywords:** Aortic stenosis, Heart failure, Aortic valve replacement

## Abstract

**Aims:**

There is a lack of robust data on the optimal medical treatment of heart failure in patients with severe aortic stenosis, with no randomized controlled trials guiding treatment. The study aimed to study the association between exposure to renin–angiotensin–aldosterone system (RAS) inhibitors or beta-blockers and outcome after aortic valve replacement in patients with aortic stenosis and heart failure.

**Methods and results:**

The study included all patients with heart failure undergoing aortic valve replacement for aortic stenosis in Sweden between 2008 and 2016 (*n* = 4668 patients). Exposure to treatment was assessed by a continuous tracking of drug dispensations, and outcome events were all-cause mortality and hospitalization for heart failure collected from national patient registries. After adjustment for age, sex, atrial fibrillation, hypertension, diabetes mellitus, and prior myocardial infarction, Cox regression analysis showed that RAS inhibition was associated with a lower risk of all-cause mortality in patients with reduced left ventricular ejection fraction (LV-EF) [hazard ratio (HR) 0.58, 95% confidence interval (CI) 0.51–0.65] and preserved LV-EF (HR 0.69, 95% CI 0.56–0.85). Beta-blockade was associated with a lower risk of all-cause mortality in patients with reduced LV-EF (HR 0.81, 95% CI 0.71–0.92), but not in preserved LV-EF (HR 0.87, 95% CI 0.69–1.10). There was no association between RAS inhibition or beta-blockade and the risk of hospitalization for heart failure.

**Conclusion:**

The RAS inhibition was associated with a lower all-cause mortality after valve replacement in patients with both reduced and preserved LV-EF. Beta-blockade was associated with lower all-cause mortality only in patients with reduced LV-EF.

## Graphical Abstract

**Graphical Abstract:**
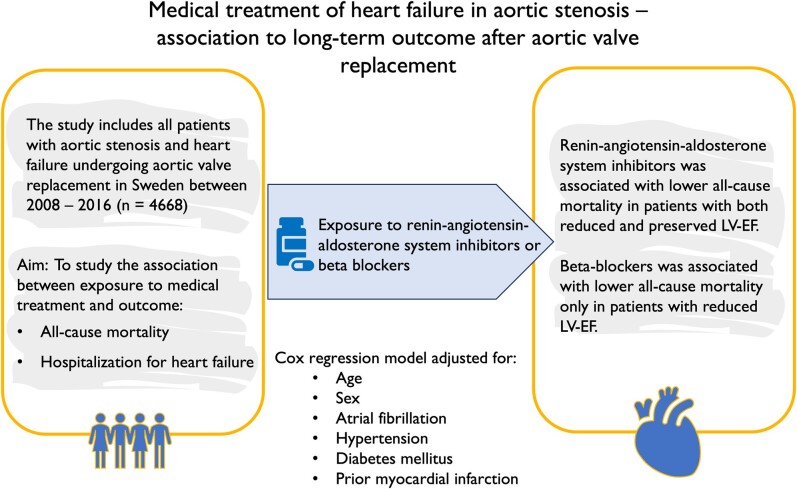

## Introduction

The natural history of aortic valve stenosis is characterized by a long, asymptomatic period of progressive valvular sclerosis, followed by a rapid clinical deterioration prompted by the development of myocardial decompensation. A narrowing of the aortic valve causes an increased strain on the ventricular wall, resulting in hypertrophy, myocardial fibrosis, and ventricular dysfunction.^1,2^ There is no precise cut-off in stenosis severity where ventricular remodulation and decompensation occur, with considerable variability between patients, making the individual assessment of the status of the left ventricular myocardium an integral part in the work-up of patients prior to valve surgery.^3,4^

Current guideline-directed treatment of severe aortic stenosis consists of aortic valve replacement in all patients who develop symptoms or signs of left ventricular decompensation.^5^ Despite valve replacement, there are still persistent left ventricular abnormalities several years after valve replacement.^6–8^ Increased mortality and hospitalization for heart failure remain a concern after valve replacement and are associated with persistent left ventricular dysfunction.^3,9,10^

There are no randomized controlled trials on the best medical treatment in patients with risk of heart failure after valve intervention in severe aortic stenosis.^11^ The renin–angiotensin–aldosterone system (RAS) is involved in the regulation of myocardial fibrosis, and RAS inhibition with angiotensin-converting enzyme inhibitors or angiotensin II receptor blockers has been shown in several studies to reverse left ventricular remodelling and left ventricular fibrosis and improve survival in patients with heart failure with reduced ejection fraction.^12–14^

Along with RAS inhibitors, beta-blockers are well established in guideline-directed medical treatment of heart failure with reduced LV-EF, and there are some observational data suggesting a possible positive effect on survival in patients with mild–moderate aortic stenosis.^15^ Standard guideline-directed medical treatment is recommended in patients with heart failure with reduced LV-EF secondary to aortic stenosis, whilst the evidence for medical treatment in patients with heart failure with preserved LV-EF is still scarce.^11^

The aim of this study was to explore the association between exposure to RAS inhibitors or beta-blockers and all-cause mortality and hospitalization for heart failure after aortic valve replacement in a national cohort of patients with severe aortic stenosis and signs of heart failure with either preserved or reduced LV-EF.

## Methods

### Study population and data sources

The study was conducted as a retrospective cohort study, and all patients were identified from the Swedish Web-system for Enhancement and Development of Evidence-based Care in Heart Disease Evaluated According to Recommended Therapies (SWEDEHEART). SWEDEHEART is a nationwide prospective, multicentre registry, where all patients undergoing aortic valve replacement in Sweden are continuously registered. The study cohort includes all patients that had undergone transcatheter or surgical aortic valve replacement (mechanical or biological) due to aortic valve stenosis, with reduced LV-EF or a diagnosis of heart failure prior to valve replacement, in Sweden between 1 January 2008 and 31 December 2016. Only patients with aortic stenosis as the primary indication for valve replacement were included in the study cohort, and patients with aortic regurgitation as the primary indication were excluded.

### Data collection

The SWEDEHEART registry contains information on the date of procedure, prosthesis type, LV-EF prior to valve replacement, cardiovascular diseases, and comorbidities. LV-EF is estimated by echocardiography prior to valve replacement and categorized in the registry as either preserved (LV-EF > 50%), mild-moderately reduced (30–50%), or severely reduced (<30%). The registry does not contain information on the exact value of LV-EF or information on which method was used to calculate the LV-EF. Outcome events, i.e. hospitalization for heart failure and all-cause mortality, were collected from the National Patient Registry (NPR) and the Swedish Cause of Death Registry (CDR). The NPR contains diagnosis codes (ICD-10) on all hospital admissions in Sweden, and the CDR contains data on the date and cause of death for all Swedish residents. Linkage was based on the unique 10-digit personal identification number assigned to all Swedish residents. The start of the follow-up was 1 day after discharge from the index valve replacement, and the study cohort was followed until death or the end of the follow-up (31 December 2016), which ever occurred first.

### Baseline information and comorbidities

Information on baseline characteristics were collected from SWEDEHEART and enriched with data from the NPR by collecting information on the ICD-10 codes for all hospital admissions up to 3 years prior to valve replacement. Predefined cardiovascular diseases and comorbidities at baseline were atrial fibrillation, hypertension, diabetes, and previous myocardial infarction. See table in the Supplementary material online for information on the selected ICD codes. The cohort included patients with heart failure separated into two groups based on LV-EF. All patients with a reduced LV-EF < 50% were included in the group with reduced LV-EF. The group with preserved LV-EF was defined as patients with an ICD code of a previous episode of hospitalization for heart failure and an LV-EF ≥ 50%.

### Exposure to renin–angiotensin–aldosterone system inhibitors and beta-blockers

Information on medical treatment was collected by computerized linkage with the Dispensed Drug Registry. The registry contains information on all prescriptions and dispensation of drugs for all Swedish residents. The exposure was defined as dispensation of a RAS inhibitor (i.e. angiotensin-converting enzyme inhibitors or angiotensin receptor blockers or a beta-blocker). Medication exposure was assessed by continuous tracking of drug dispensations after the index valve replacement. The patient was considered exposed to treatment for 120 days after each dispensation of a prescription of the specific drug. Information on dispensation was continuously updated, and patients could change groups during follow-up according to exposure. The population was then stratified according to exposure to RAS inhibitor and beta-blocker, with patients exposed to either RAS inhibitor or beta-blocker after valve replacement constituting the exposed group and patients who were not prescribed treatment constituting the non-exposed control group.

## Outcome events

Outcome events after discharge from the index valve replacement were all-cause mortality and hospitalization for heart failure. Hospitalization for heart failure was only considered an outcome event when it was the main diagnosis of the admission.

### Statistical methods

Categorical data were presented as frequencies and percentages and continuous data as median and interquartile range (Q1–Q3). The χ^2^ test was used for comparison between categorical variables.

Exposure to treatment with the RAS inhibitor and beta-blockers was updated at every dispensation, and the total exposure time to each treatment was calculated as the total person-time spent on either a RAS inhibitor or a beta-blocker. It was reported in person-years of exposure to treatment, and the incidence rate in events per 100 person-years was calculated.

Kaplan–Meier curves for all-cause mortality and hospitalization for heart failure occurring before the end of the follow-up (31 December 2016) were created by restarting the patients after a treatment switch. Patients were censored for the endpoint hospitalization for heart failure when deceased.

The association between exposure to drug treatment and outcome events was analysed using both unadjusted and adjusted Cox regression models. In the adjusted model, confounders were chosen based on the assumed clinical relevance and consisted of age, sex, atrial fibrillation, hypertension, diabetes mellitus, and prior myocardial infarction. Due to the lack of robust data on kidney function, it was not included as a confounder in the adjusted Cox regression model. Analyses were performed separately for patients with preserved and reduced LV-EF. The results of the unadjusted and adjusted Cox proportional hazard models were presented as HRs with 95% CIs. An HR < 1 corresponds to a lower risk when treated. Hospitalization for heart failure has repeated events. Thus, if a patient experiences several readmissions for heart failure during follow-up, all of them are taken into account. Age was modelled as a four-knot restricted cubic spline to handle possible non-linearities. We also performed a subgroup analysis of patients treated with RAS inhibitors or beta-blockers 4 months prior to valve replacement.

Statistical analyses of baseline characteristics were performed with a commercially available software package (SPSS Statistics 28, SPSS Inc., Chicago, IL). All other statistical analyses were performed in R version 4.2.1.

## Results

### Baseline characteristics

A total of 4668 patients were identified and included in the study cohort, 3775 with reduced LV-EF and 913 with preserved LV-EF. The median age at intervention was 75 years (IQR 68–81) for patients with reduced LV-EF and 79 years (IQR 72–84) for those with preserved LV-EF. Amongst patients with reduced LV-EF, 1057 (28.1%) were female in comparison with 495 (54.2%) patients with preserved LV-EF. In patients with reduced LV-EF, the majority had mild to moderately reduced LV-EF between 30% and 50%, and 770 patients (20.5%) had severely reduced LV-EF < 30%. Patients with preserved LV-EF had a higher proportion of hypertension and atrial fibrillation than patients with reduced LV-EF, The proportion of diabetes mellitus and prior myocardial infarction was similar in both groups, as presented in *Table 1*.

**Table 1:** Baseline characteristics expressed as frequencies and percentages if not otherwise stated

	Reduced LV-EF	Preserved LV-EF	Total
	*n* = 3775	*n* = 913	*n* = 4668
Age, median (Q1–Q3)	75 (68–81)	79 (72–84)	76 (69–82)
Gender			
Female (%)	1057 (28.1)	495 (54.2)	1552 (33.2)
Male (%)	2698 (71.9)	418 (45.8)	3116 (66.8)
Prosthesis type			
SAVR mechanical (%)	427 (11.4)	48 (5.3)	475 (10.2)
SAVR biological (%)	2420 (64.4)	481 (52.7)	2901 (62.1)
TAVR (%)	908 (24.2)	384 (42.1)	1292 (27.7)
LV-EF			
>50% (%)	0 (0.0)	913 (100)	913 (19.6)
30–50% (%)	2985 (79.5)	0 (0.0)	2985 (63.9)
<30% (%)	770 (20.5)	0 (0.0)	770 (16.5)
Comorbidities			
Hypertension (%)	1917 (51.1)	652 (71.4)	2569 (55.0)
Diabetes mellitus (%)	1030 (27.4)	252 (27.6)	1282 (27.5)
Previous myocardial	860 (22.9)	183 (20.0)	1043 (22.3)
Infarction (%)			
Atrial fibrillation (%)	1207 (32.1)	454 (49.7)	1661 (35.6)

SAVR, surgical aortic valve replacement; TAVR, transcatheter aortic valve replacement; LV-EF, left ventricular ejection fraction.

### Outcome events during follow-up

The median follow-up was 3.6 years in the group with reduced LV-EF and 3.4 years in the group with preserved LV-EF. Exposure to drug treatment during follow-up is presented in *Table 3*. There were a total of 1516 deaths during follow-up, with a higher incidence rate in patients with preserved LV-EF (10.4 events/100 person-years) compared with those with reduced LV-EF (7.5 deaths/100 person-years). There were a total of 2013 hospitalizations for heart failure, with a higher incidence rate in patients with preserved LV-EF (14.8 events/100 person-years) than in patients with reduced LV-EF (9.7 events/100 person-years), as presented in *Table 2*.

**Table 2:** Outcome events during follow-up showing number of events, total follow-up time in person-years, and incidence rate, i.e. events per 100 person-years

Outcome	Number of events	Person-time	Incidence rate
All-cause mortality			
Reduced LV-EF	1148	15 400	7.5
Preserved LV-EF	368	3520	10.4
Hospitalization for heart failure			
Reduced LV-EF	1490	15 400	9.7
Preserved LV-EF	523	3520	14.8

LV-EF, left ventricular ejection fraction.

**Table 3:** Exposure to drug treatment during follow-up in person-time in years spent on each drug (percentage of total person-time)

Drug	Reduced LV-EF	Preserved LV-EF
RAS inhibitor (%)	9919 (64.4)	2082 (59.1)
Beta-blocker (%)	11 590 (75.3)	2674 (75.9)

LV-EF, left ventricular ejection fraction.

### All-cause mortality and treatment with renin–angiotensin–aldosterone system inhibitor or beta-blocker

**Figure 1:**
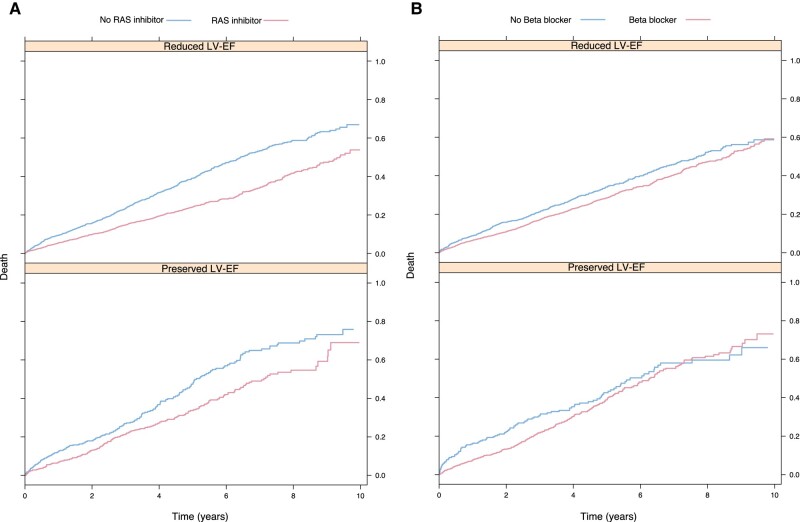
(*A*) Kaplan–Meier plots showing all-cause mortality by exposure to a RAS inhibitor in patients with reduced and preserved LV-EF. LV-EF, left ventricular ejection fraction; RAS inhibitor, renin–angiotensin–aldosterone system inhibitor. (*B*) Kaplan–Meier plots showing all-cause mortality by exposure to beta-blockers in patients with reduced and preserved LV-EF. LV-EF, left ventricular ejection fraction.

**Figure 2:**
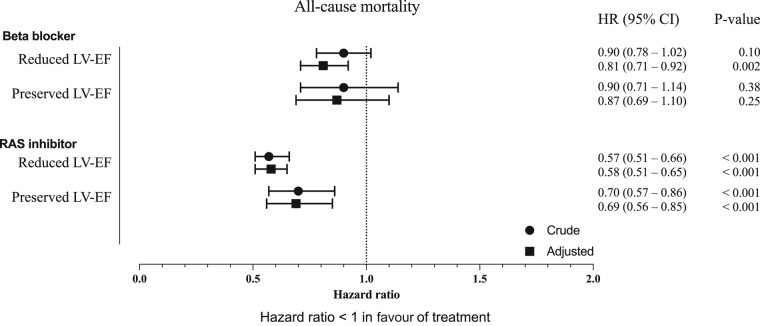
Forest plot with hazard ratios (HRs) from crude and adjusted Cox models for the risk of all-cause mortality after valve replacement by exposure to RAS inhibitor or beta-blocker therapy in patients with reduced and preserved LV-EF respectively. Also presented is the 95% confidence interval (CI) for each HR and respective *P* value. An HR < 1 corresponds to a lower risk when exposed to the specific drug. The adjusted Cox model was adjusted for age, gender, hypertension, diabetes mellitus, atrial fibrillation, and prior myocardial infarction. LV-EF, left ventricular ejection fraction; RAS inhibitor, renin–angiotensin–aldosterone system inhibitor.

All-cause mortality was lower in patients exposed to the RAS inhibitor compared with patients without the RAS inhibitor, in both patients with reduced and preserved LV-EF (*Figure 1A*). After adjustment, the RAS inhibitor was associated with a lower risk of all-cause mortality (HR 0.58, 95% CI 0.51–0.65) in patients with reduced LV-EF. A similar result was found in patients with preserved LV-EF (HR 0.69, 95% CI 0.56–0.85), as presented in *Figure 2*. In a subgroup analysis of patients treated with RAS inhibitors prior to valve replacement, exposure to RAS inhibition after valve replacement shows similar results (Supplementary material online, Table *S1A*).

All-cause mortality was lower in patients exposed to beta-blockers compared with patients without beta-blockers (*Figure 1B*). After adjusting for confounders, the HR for all-cause mortality was 0.81 (95% CI 0.71–0.92) in patients with reduced LV-EF exposed to beta-blockers compared with those not exposed. After adjustment, there was no association between beta-blockers and all-cause mortality in patients with preserved LV-EF (*Figure 2*). In a subgroup analysis of patients treated with beta-blockers prior to valve replacement, exposure to beta-blockers after valve replacement shows similar results as the main analysis (Supplementary material online, Table *S1B*).

### Hospitalization for heart failure and treatment with renin–angiotensin–aldosterone system inhibitor or beta-blocker

**Figure 3:**
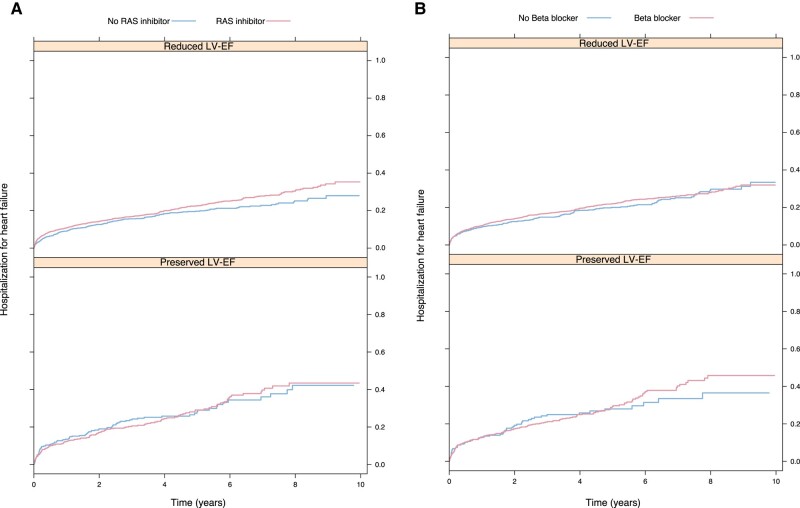
(*A*) Kaplan–Meier plots showing hospitalization for heart failure by exposure to RAS inhibitor in patients with reduced and preserved LV-EF. LV-EF, left ventricular ejection fraction; RAS inhibitor, renin–angiotensin–aldosterone system inhibitor. (*B*) Kaplan–Meier plots showing hospitalization for heart failure by exposure to beta-blockers in patients with reduced and preserved LV-EF. LV-EF, left ventricular ejection fraction.

**Figure 4:**
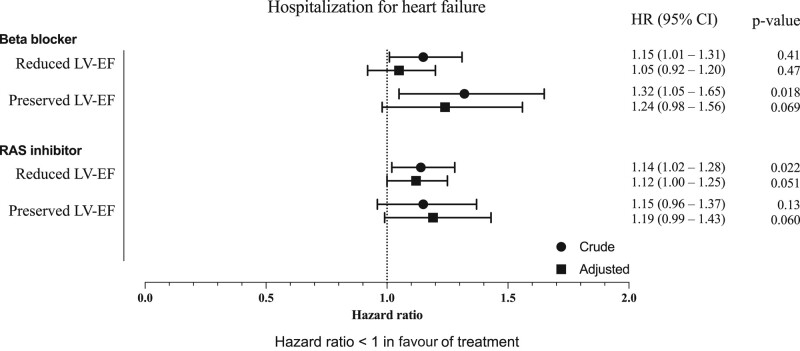
Forest plot with hazard ratios (HR) from crude and adjusted Cox models for the risk of hospitalization of heart failure after valve replacement by exposure to RAS inhibitor or beta-blocker therapy in patients with reduced and preserved LV-EF. Also presented is the 95% confidence interval (CI) for each HR and respective *P* value. An HR < 1 corresponds to a lower risk when exposed to the specific drug. The adjusted Cox model was adjusted for age, gender, hypertension, diabetes mellitus, atrial fibrillation, and prior myocardial infarction. If patients experienced several hospitalizations for heart failure during follow-up, all of them were taken into account. LV-EF, left ventricular ejection fraction; RAS inhibitor, renin–angiotensin–aldosterone system inhibitor.

The frequency of hospitalization for heart failure separated by the RAS inhibitor and beta-blockers in patients with reduced and preserved LV-EF is described in *Figure 3A* and *3B*. There was no significant association between exposure to the RAS inhibitor and the risk of hospitalization of heart failure in patients with reduced LV-EF (HR 1.12, 95% CI 1.00–1.25) or preserved LV-EF (HR 1.19, 95% CI 0.99–1.43), as presented in *Figure 4*. In a subgroup analysis of patients treated with the RAS inhibitor prior to valve replacement, exposure to RAS inhibition showed a lower risk of hospitalization for heart failure in patients with heart failure and reduced LF-EF, whilst results similar to the main analysis were found for patients with preserved LV-EF (Supplementary material online, Table *S1A*).

There was no significant association between exposure to beta-blocker and the risk of hospitalization for heart failure in patients with reduced LV-EF (HR 1.05, 95% CI 0.92–1.20). Similar results were found in patients with preserved LV-EF (HR 1.24, 95% CI 0.98–1.56), as presented in *Figure 4*. In the subgroup analysis of patients treated with beta-blockers prior to valve replacement, similar results were found (Supplementary material online, Table *S1B*).

## Discussion

The main finding from this retrospective cohort study of patients with aortic stenosis undergoing aortic valve replacement is the significant association between exposure to RAS inhibitors and a lower risk of all-cause mortality after valve replacement in all patients with heart failure and with both reduced and preserved LV-EF. Exposure to beta-blockers after valve replacement is significantly associated with lower all-cause mortality in patients with reduced LV-EF, but not in patients with preserved LV-EF.

Although randomized controlled trials on the effects of RAS inhibition on clinical outcomes in patients with severe aortic stenosis are lacking, left ventricular hypertrophy and myocardial fibrosis are commonly observed in patients with severe aortic valve stenosis, making RAS inhibition a potentially promising therapeutic target.^2,8^ In the absence of randomized trials, data from observational studies suggest a possible survival benefit of RAS inhibitors after aortic valve replacement in severe aortic stenosis.^11,16–18^ The findings in this study are in line with the rationale that RAS inhibitors could potentially improve reverse remodelling after valve replacement and improve long-term prognosis, suggesting a potential role for RAS blockade in patients with severe aortic stenosis and signs of left ventricular decompensation, regardless of LV-EF. The results also confirm the association between beta-blockers and improved survival in patients with reduced LV-EF, but not in preserved LV-EF.

A strength of the current study is the continuous tracking of drug dispensations after valve replacement, ensuring good data on treatment exposure at the time of valve replacement as well as during follow-up. Whilst showing an association with survival, the main analysis did not show a reduced risk in hospitalization for heart failure in patients with neither reduced nor preserved LV-EF treated with RAS inhibitors or beta-blockers after valve replacement, although a subgroup analysis did show a reduced risk of hospitalization for heart failure in patients with reduced LV-EF that were treated with RAS inhibitors prior to valve replacement and continued treatment after valve replacement. One factor that could potentially explain part of the lack of association between treatment and risk of hospitalization for heart failure could be timing of treatment. In a recent observational study on patients with severe aortic stenosis doing transcatheter aortic valve replacement, preoperative treatment with RAS inhibitor was associated with a significantly lower risk of mortality compared with postoperative treatment only. The authors did not, however, specifically study the association between preoperative treatment and risk for hospitalization for heart failure, but the question of optimal timing of treatment warrants further research.^19^ Several studies have shown that whilst left ventricular hypertrophy and diffuse interstitial fibrosis regress after valve replacement, replacement fibrosis does not and has been shown to negatively impact long-term prognosis and increase the risk of heart failure decompensation after valve replacement.^7,8,20^ There is a need for more robust data on the effects of treatment with RAS inhibitors during the earlier stages of the disease to learn whether it could affect the development of replacement fibrosis and reduce the risk of developing heart failure.

As an observational study, there are several limitations, and caution should be taken in interpreting the results. Despite adjusting for several important confounding variables, there may still exist residual confounders affecting the outcome. Due to the lack of randomization, there is a risk of selection bias that could affect the association between treatment and risk of hospitalization for heart failure. As patients who are more overtly symptomatic may be more likely to be prescribed heart failure medications, they might also be more likely to present with heart failure symptoms during follow-up. Such a selection bias could possibly attenuate the treatment effect of RAS inhibitors and beta-blockers on the risk of hospitalization for heart failure. Other possible confounders in this material are the degree of kidney failure in patients included in the cohort as we lack robust data on kidney function and have not been able to adjust for this confounder in our Cox regression models. We also lack data on AV gradients and prevalence of aortic regurgitation after valve replacement, which are factors that could possibly have affected the outcomes.

When interpreting the association between treatment exposure and hospitalization for heart failure in our study population, one also needs to be aware of the possible fallacies of using ICD codes to monitor the outcome. To ensure the validity of the diagnosis, we have only included admissions where heart failure was the primary diagnosis, and there is a risk of underestimating the burden of heart failure in our study population.^21,22^ Another factor that might influence sensitivity is an increasing trend towards outpatient care of heart failure as not all patients with worsening heart failure symptoms are hospitalized for heart failure and thus not included in the outcome data.^23^

As we learn more of the many different phenotypes of heart failure in valve disease, we are also learning that one size does not fit all in the way of heart failure treatment. This study adds to the growing body of evidence of a possible survival benefit of RAS inhibition in patients with severe aortic stenosis by showing a possible survival benefit, regardless of LV-EF prior to valve replacement in both surgical and transcatheter valve replacements. It also prompts further research into the optimal timing of treatment with RAS inhibition.

There are no randomized trials and limited observational data on the association between RAS inhibitors or beta-blockers and the risk of hospitalization for heart failure after aortic valve replacement. The available observational data are conflicting, with some studies showing a possible association between RAS inhibitors and the lower risk of hospitalization for heart failure after aortic valve replacement while other studies have failed to show a clear association.^17,24^ Considering the risk of confounding, the results need to be interpreted with caution. We need more robust data from randomized controlled trials before conclusions can be drawn as to the effect of RAS inhibitors and beta-blockers on survival and the risk of hospitalization for heart failure after valve replacement, especially in patients with preserved LV-EF, where evidence of effective treatment alternatives is still scarce.

## Lead author biography


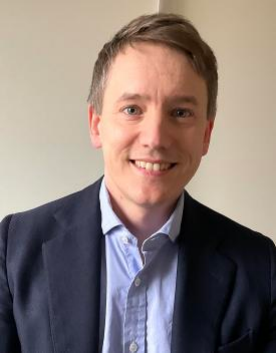


Dr Johan Hopfgarten is a consultant cardiologist at Uppsala University Hospital, Uppsala, Sweden. He completed his medical degree at Uppsala University and is currently a PhD candidate at the Department of Medical Sciences at Uppsala University studying left ventricular dysfunction in aortic stenosis.

## Supplementary Material

oeae039_Supplementary_Data

## Data Availability

SWEDEHEART does not allow individual data sharing to third parties. Access to aggregated data might be granted following review by the SWEDEHEART steering committee. Such requests can be submitted to the SWEDEHEART steering committee for consideration. For contact details, see https://www.ucr.uu.se/swedeheart/kontakt/support.
