# Supplementary material for: Medical treatment of heart failure with renin–angiotensin–aldosterone system inhibitors and beta-blockers in aortic stenosis: association with long-term outcome after aortic valve replacement
Source: Eur Heart J Open. 2024 May 9;4(3):oeae039. doi: 10.1093/ehjopen/oeae039 (PMC11135942; doi:10.1093/ehjopen/oeae039)
Supplement: oeae039_Supplementary_Data [file oeae039_supplementary_data.docx]

| *Supplement table 1a* | | | | | | | | |
| --- | --- | --- | --- | --- | --- | --- | --- | --- |
|  |  |  | Crude | | Adjusted | | | |
|  |  | HR | | 95% CI | |  | HR | 95% CI |
| *All-cause mortality* | *Reduced LV-EF* | 0.43 | | 0.36 - 0.51 | |  | 0.46 | 0.39 - 0.55 |
|  | *Preserved LV-EF* | 0.64 | | 0.47 - 0.87 | |  | 0.58 | 0.42 - 0.79 |
|  |  |  | |  | |  |  |  |
| *Hospitalization for heart failure* | *Reduced LV-EF* | 0.67 | | 0.57 - 0.78 | |  | 0.74 | 0.63 - 0.86 |
|  | *Preserved LV-EF* | 1.09 | | 0.81 - 1.48 | |  | 1.07 | 0.78 - 1.45 |
| The table presents results from crude and adjusted Cox models for all-cause mortality and hospitalization for heart failure after valve replacement in a subgroup analysis of patients exposed to RAS inhibitor at a time point four months prior to valve replacement. The tables show hazard ratios for continued treatment with RAS inhibitor after valve replacement and a HR < 1 correspond to lower risk when treated. The results are presented for patients with reduced or preserved LV-EF respectively. Also presented is the 95% confidence interval (CI) for each HR. The adjusted Cox model was adjusted for age, sex, hypertension, diabetes mellitus, atrial fibrillation and prior myocardial infarction.  Footnote: LV-EF, left ventricular ejection fraction; RAS inhibitor, renin-angiotensin-aldosterone system inhibitor. | | | | | | | | |

| *Supplement table 1b* | | | | | | | | |
| --- | --- | --- | --- | --- | --- | --- | --- | --- |
|  |  |  | Crude | | Adjusted | | | |
|  |  | HR | | 95% CI | |  | HR | 95% CI |
| *All-cause mortality* | *Reduced LV-EF* | 0.80 | | 0.68 - 0.94 | |  | 0.74 | 0.63 - 0.87 |
|  | *Preserved LV-EF* | 0.73 | | 0.56 - 0.96 | |  | 0.73 | 0.55 - 0.96 |
|  |  |  | |  | |  |  |  |
| *Hospitalization for heart failure* | *Reduced LV-EF* | 1.00 | | 0.84 - 1.18 | |  | 0.94 | 0.79 - 1.11 |
|  | *Preserved LV-EF* | 1.23 | | 0.92 - 1.65 | |  | 1.18 | 0.88 - 1.57 |
| The table presents results from crude and adjusted Cox models for all-cause mortality and hospitalization for heart failure after valve replacement in a subgroup analysis of patients exposed to beta blockers at a time point four months prior to valve replacement. The tables show hazard ratios for continued treatment with beta blockers after valve replacement and a HR < 1 correspond to lower risk when treated. The results are presented for patients with reduced or preserved LV-EF respectively. Also presented is the 95% confidence interval (CI) for each HR. The adjusted Cox model was adjusted for age, sex, hypertension, diabetes mellitus, atrial fibrillation and prior myocardial infarction.  Footnote: LV-EF, left ventricular ejection fraction | | | | | | | | |
